# ASD: Biochemical Mechanisms behind Behavioral Disorders

**DOI:** 10.1155/2014/758473

**Published:** 2014-03-27

**Authors:** Giuseppe Valacchi, Paul Ashwood

**Affiliations:** ^1^Department of Life Science and Biotechnologies, University of Ferrara, Via Borsari 46, 44100 Ferrara, Italy; ^2^Department of Medical Microbiology and Immunology, and the MIND Institute, University of California, UC Davis Sacramento, Davis, CA 95817, USA

Autism spectrum disorders (ASD) are complex neurobiological disorders of development characterized by impairments in social interaction and communication, together with restricted, repetitive, and stereotyped interests/behavior. There has been a dramatic increase in the reported rates of ASD over the last 40 years which has risen in USA from 1 in 5000 in the mid-1970s to 1 in 88 in 2012. However, little is known about the underlying pathophysiological mechanisms and there is currently a lack of reliable biological markers to help in the diagnosis or monitoring of the changes in clinical definitions over time or in response to therapies. Currently, there are few effective treatments for ASD with most medication and behavioral therapies aimed at minimizing the symptomology. Although the knowledge base of ASD is rapidly growing as research examines more and varied aspects of these disorders, their complex nature makes it difficult to determine the causation or catalysts. There is probably no singular unique cause for these neurobehavioral disorders but a combination of genetic and environmental factors may be responsible for pathological changes in brain and immune, metabolic, and endocrine systems.

The present special issue collects reports related to the possible pathological mechanisms involved in ASD with special focus on classic autism and Rett syndrome (RTT). Growing evidence suggests that immune dysfunction and the presence of autoimmune responses including autoantibodies may play a role in ASD. The article by M. Careaga et al. provides evidence that young children with ASD have an elevated production of anti-phospholipid antibodies. The authors have shown an increased level of anticardiolipin, *β*2-glycoprotein 1, and anti-phosphoserine antibodies and this increase was associated with more impaired behaviors of the patients. In line with Careaga's work, a paper by C. Giulivi et al. investigated the maternal immune activation (MIA) as a potential risk factor for ASD and schizophrenia in a mouse model. The authors showed that splenocytes isolated from adult offspring exposed gestationally to the viral mimic poly(I:C) (to activate MIA) had long-lasting effects in the bioenergetics with a significant reduction of ATP production as a consequence of lower mitochondrial complex I activation. The work by L. Ciccoli et al. showing an unrecognized triad combination of erythrocyte shape abnormalities, erythrocyte membrane oxidative damage, and *β*-actin alterations in individuals with ASD provides, therefore, a new possible biological marker for the diagnosis of ASDs.

In the second part of the special issue, the reports are related to several clinical and biochemical aspects present in Rett syndrome (RTT). Of interest is the paper by A. Pecorelli et al. where the authors have analysed the genome expression of limphomonocytes isolated from RTT patients showing altered gene expression related to mitochondrial function, cellular ubiquitination, proteasome degradation, RNA processing, and chromatin folding, suggesting, therefore, that mitochondrial-ATP-proteasome function is likely to be involved in RTT clinical features. In addition, the work by C. De Felice et al. has shown that pulmonary gas exchange abnormality (GEA) is present in RTT and that terminal bronchioles are a likely major target of the disease. There are a further 2 papers relating to the positive effect of *ω*-3 treatment in RTT. The first by S. Maffei et al. shows that**ω**-3 PUFAs are able to improve the biventricular myocardial systolic function in those patients and that the functional gain is partially mediated through a regulation of the redox balance. The other, by C. De Felice et al., shows that *ω*-3 PUFAs supplementation is able to partially rescue the acute phase response present in RTT. This last result is in line with the paper by A. Cortelazzo et al. where, through a proteomic approach, the authors were able to demonstrate the presence of a subclinical chronic inflammatory status related to the severity carried by the* MECP2 *gene mutation. Finally, again utilizing a proteomic approach, A. Cortelazzo et al. were able to compare the proteasome expression of classical RTT versus speech variant RTT in 2 sisters. They suggest that unique familial cases offer the opportunity to identify new protein patterns involved in the RTT phenotype expression.

Taken together, these publications offer new light on the pathophysiology of ASD and Rett syndrome and the potential to further investigate biological markers and possible therapeutic approaches.

*Giuseppe Valacchi* *Giuseppe Valacchi* *Paul Ashwood* *Paul Ashwood*

